# Experience, facilitators, and barriers to the implementation of a multicomponent programme in older people living in the community, +AGIL Barcelona: A qualitative study

**DOI:** 10.3389/fpubh.2023.1161883

**Published:** 2023-03-30

**Authors:** Olga Canet-Vélez, Lilian Solis-Navarro, Mercè Sitjà-Rabert, Laura Mónica Pérez, Judith Roca, Luis Soto-Bagaria, Rodrigo Torres-Castro, Francisco Díaz-Gallego, Jordi Vilaró, Marco Inzitari

**Affiliations:** ^1^Facultat Ciències de la Salut Blanquerna, Universitat Ramon Llull, Barcelona, Spain; ^2^Research Group Global Health, Gender and Society (GHenderS), Barcelona, Spain; ^3^Programa de Doctorat-Facultat Ciències de la Salut Blanquerna, Universitat Ramon Llull, Barcelona, Spain; ^4^Global Research on Wellbeing (GRoW), Barcelona, Spain; ^5^Research on Aging, Frailty and Care Transitions in Barcelona (REFiT-BCN), Parc Sanitari Pere Virgili and Vall d'Hebron Institute (VHIR), Barcelona, Spain; ^6^Department of Nursing and Physiotherapy, University of Lleida, Lleida, Spain; ^7^Health Care Research Group (GRECS), Biomedical Research Institute of Lleida, Lleida, Spain; ^8^Health Education, Nursing, Sustainability and Innovation Research Group (GREISI), University of Lleida, Lleida, Spain; ^9^Department of Physical Therapy, University of Chile, Santiago, Chile; ^10^Institut Català de la Salut, Gerència de Barcelona, Barcelona, Spain; ^11^Primary Healthcare Center Bordeta-Magòria, Institut Català de la Salut, Barcelona, Spain; ^12^Faculty of Health Sciences and eHealth Center, Universitat Oberta de Catalunya (UOC), Barcelona, Spain

**Keywords:** frailty, community-integrated care, exercise implementation programme frailty, exercise implementation programme, exercise

## Abstract

**Introduction:**

The +AGIL Barcelona programme is a multicomponent care intervention for frail older adults (FOAs) living in the community. To improve the programme, it is essential to investigate the experience of all participants. Our objective was to explore the perspective of FOA and professionals about the barriers, facilitators, and improvement elements of the development of the +AGIL Barcelona programme. Qualitative descriptive approach. Were included FOA and professionals who participated in the +AGIL Barcelona programme.

**Methods:**

Three focus groups and four interviews were conducted. These were analyzed following the qualitative method of content analysis. The criteria of scientific rigor of credibility, dependence, and transferability were ensured throughout the study.

**Results:**

Three themes and seven sub-themes were developed: facilitators (positive experience and perceived benefits), barriers (self-perceived health status, digital divide, and continuity of the programme at home), and improvements elements (programme continuity and adaptation of technology). All the participants felt satisfied, highlighting aspects such as interpersonal relationships and social contact, face-to-face sessions guided by a physiotherapist, and the functional improvement achieved. Some of the difficulties were the self-perception of frailty, the need for technological support, and continuing the exercise programme at home.

**Conclusion:**

The FOA who participated in the +AGIL Barcelona programme perceived direct benefits for their health and physical condition due to the development of self-confidence by being able to perform physical exercise despite their baseline condition, and the professionals experienced an improvement in the quality of care due to work in a multidisciplinary team.

## Introduction

Aging population is a global reality that requires adapting and integrating different levels and models of care to provide appropriate healthcare to this group's needs and specific characteristics (1). In addition, the coordination and integration of frailty programmes for the older adult population are challenges that aging entails in our society.

The concept of frailty has evolved since Linda Fried described the physical frailty phenotype (2). Frailty is currently defined as a state of vulnerability, potentially reversible, to internal and/or external stressors (3). This state entails adverse health effects such as functional impairment, hospitalization, disability, institutionalization, increased morbidity and mortality, and increased health expenditure (4). In Europe, in 2019, the prevalence of physical frailty was 15% in people over 65 living in the community (5).

There is consensus and robust evidence on managing frailty with strategies based on geriatric assessment and multifactorial interventions where multicomponent physical exercise, nutrition, management of polypharmacy, and health education are the central axis of the programmes (6, 7). Older people believe in the potential of physical activity to improve their physical and mental states. However, it is essential to consider that lack of social support, previous sedentary habits, difficulties in accessing programmes, and apathy are significant barriers to the participation and adherence of older people in exercise programmes (8, 9).

Current research on the experience of frail older adults (FOAs) and professionals in implementing physical exercise programmes is almost non-existent (10). However, a study indicates that the accessibility to the environment where the exercise is carried out is closely related to the security perceived by the frail older person (10). Furthermore, it should be considered that frailty is accompanied by reduced physical strength, slow mobility, vision problems, and fatigue, which are perceived as a risk for the individual (10). Thus, to successfully implement these programmes, shared decision-making between the professional and the older adult is essential to balance risks and benefits. In particular, finding an intrinsic motivation allows for identifying objectives that frail adults can achieve in their practice of physical exercise (11–14).

The +AGIL Barcelona programme is a multicomponent care intervention for FOA living in the community, building on a comprehensive care model that involves primary, geriatric, and community care. Its results show clinically and statistically significant improvement in physical function and gait speed in patients with different degrees of initial frailty (15). In the last years, given the restrictions imposed by COVID-19 and to improve its scalability, the digital component of the programme was incremented (16). The use of digital technologies could be a motivation for FOA to perform physical exercise. Even so, it has been observed that, regardless of age, educational level, or opinion regarding technology, frailty is a condition that leads to less use of digital technology (17). Furthermore, the successful use of digital tools in health promotion programmes for older adults highly depends on the motivation and support they receive when using these tools (18).

For this reason, the main objective of this study was to explore the perspective of FOA and professionals about the barriers, facilitators, and improvement elements of the development of the +AGIL Barcelona programme, including its digital component.

## Methods

### Design

The research design was a qualitative descriptive whose objective was to describe the phenomenon and its characteristics through the participants (19).

### Context and participants

This qualitative study was part of the +AGIL Barcelona, “A community programme of integrated care for FOA” (15). The programme is carried out due to the collaboration between a specialized geriatric team (GT) and a primary healthcare team (PHC). The PHC consists of a primary care doctor and a nurse who identify potential participants, refer them to the GT, and do subsequent follow-ups. The GT includes a geriatrician (who assesses the participants and proposes them a tailored multifactorial intervention) and a physiotherapist (who once a week performs an exercise session). Both teams provided advice and health education in the primary healthcare center (PHCC) and facilitated continuity with available resources in the community.

The GT performs a comprehensive geriatric assessment and a frailty status evaluation. Based on the results, a multifactorial intervention is planned and proposed. It includes pharmacological treatment adequacy, counseling, health education, detection and management of cognitive impairment or loneliness, and a physical exercise programme, supervised by the physiotherapist in 10 one-hour group sessions per week, with individualized dosing for each participant in terms of type, intensity, and progression of multicomponent exercises. In addition, the exercise component is complemented by strategies to increase the participant's empowerment, adherence, and continuity of exercise in the community and by the Vivifrail app, a publicly available digital application providing an exercise programme tailored to an initial auto-evaluation of physical function (15).

Our study was recruited using convenience criteria such as feasibility, access, interest, and time until data saturation was reached (20). The sample consisted of 22 participants: 11 end users and 11 professionals from the health and social sector. The inclusion criteria were as follows: (1) users with willingness and cognitive ability to express their experience that they are doing at least half of the programme at the time of the interview or have recently completed the programme and (2) health and social professionals (general practitioner, nurse, physiotherapist, civic center manager, healthcare manager, and geriatrician) involved with the +AGIL Barcelona programme. There were no exclusion criteria.

All participants were invited personally or by email and informed about the objective and content of the present study, the data collection methods, and the need to sign the informed consent for data collection.

### Data collection

Information was collected using focus groups and individual interviews to ensure the triangulation and saturation of information from October to December 2021. Three focus groups (two FOA and one professional) and four interviews (one FOA and three professionals) were conducted. Focus groups were implemented, keeping groups separate between FOAs and professionals to maximize the comfort of all participants to express their experiences and opinions. The script protocol for the interviews and focus group was evaluated by two experts in methodology and two in the subject of study. Table 1 presents an outline of the areas explored and the questions used.

**Table 1:** Research questions.

**Area**	**Research questions for frail older adults**	**Research questions for the health and social professionals**
Overall programme (facilitators and barriers)	1. How have you experienced the programme in general? How did you feel during the sessions?	1. What overall assessment do you make of implementing the intervention?
	2. Once all the sessions were finished, what has been your experience? What aspects would you highlight?	2. What aspects would you highlight?
	3. What positive things would you highlight about the physical exercise programme? What has motivated you or would encourage you to continue doing the sessions?	3. What components were perceived as facilitators during the intervention and what elements could be included to favor or support the intervention?
	4. What barriers (problems, trouble) occurred during the implementation the +AGIL Barcelona programme?	4. What barriers occurred during the implementation of the +AGIL Barcelona programme?
Use of technologies	5. In the use of “technology,” how has your experience been? What did you think?	5. What barriers did you perceive in the participants? What was the main barrier perceived by you in the participants?
	6. If you could choose, what would be your preferences related to the devices (Apps) and the type of sessions (face-to-face, online, and group)?	6. How could the digital divide be minimized in order to improve implementation?
	7. What would help or motivate you to use them more? (Technological aids)	7. Would some aspects will enhance the use of digital resources?
Improvement	8. We would like you to help us improve this programme. What changes could be made? How do you think it would be better for you?	8. From the different moments of implementation, what aspects of improvement could be incorporated? (design/planning, intervention, and evaluation)
	9. Would you incorporate anything that we haven't thought of? Would you remove any?

Three researchers (MSR, OC, and LSN) conducted the focus groups and individual interviews in pairs, with one researcher moderating and another assisting and taking field notes. All of them were audio and/or video recorded.

The interviews lasted between 30 and 45 min, and the focus groups were between 45 and 90 min. The privacy of the participants was ensured through pseudonymisation. The FOA focus groups were held in spaces provided by the primary healthcare center, ensuring their confidentiality. The focus groups were face-to-face and were complemented by an interview. As for the professionals, due to the COVID-19 safety measures, the focus groups and the interviews were carried out online. Transcripts were distributed to all the participants, and they accepted the transcript's contents.

In addition, all participants' basic sociodemographic data (age and gender) were collected. The professionals were asked about their basic training and years of experience. For the characterization of the FOAS, data were extracted from the baseline comprehensive geriatric assessment. These data were as follows: functional ability measured with the Barthel and Lawton–Brody index; physical performance measured with short physical performances battery (SPPB); frailty status measured with the Rockwood clinical frailty scale (CFS); cognitive state measured with Mini-Cog test; polypharmacy understood as the chronic use of at least five drugs or more and falls in the last year.

### Data analysis

Content analysis was used, developing the phases of preparation of the transcripts, analysis with the coding of units of meaning, and grouping into categories and thematic axes (21). This process was carried out with the support of ATLAS-ti program version 9.

The analysis of focus group discussions and individual interviews was carried out independently by two researchers (OC and JR). Once finished, the codes, categories, and themes were unified and agreed upon.

The criteria of scientific rigor proposed by Graneheim and Lundman (21) and Graneheim et al. (22) of credibility, dependence, and transferability were ensured throughout the study. The checklist of COREQ qualitative designs was used to execute and evaluate the study (23).

### Ethical considerations

This study was approved by the Clinical Research Ethics Committee of the Institut Universitari d'Investigació en Atención Primaria, Jordi Gol. Informed consent was requested from the participants. Confidentiality and anonymity in data processing were guaranteed.

## Results

The main characteristics of the participants are presented in Table 2. The mean age of FOA was 83 years, the majority were female population (91%) and 70% had over 3 points in the Mini-Cog test. A total of 78% of participants were vulnerable to being moderately fragile, 45% had falls in the last year, and 82% had polypharmacy. The professionals were also mostly women (82%), with a high experience of 15 years (SD = 4). The most prevalent profession was a doctor: two general practitioners (GPs) and two geriatricians, followed by three physiotherapists.

**Table 2:** Description of participants.

**Frail older adults characteristics**	***N* = 11**
Age, years (mean ± SD)	83 ± 5
**Sex**	
Female (*n*)	10
Male (*n*)	1
Living alone	4
**Functional ability (median** ±**IQR)**	
Barthel index	95 (73.8–98.8)
Lawton-Brody	7.5 (1.8–8)
**Physical performance (mean** ±**SD)**	
SPPB score	6.4 ± 3.1
**Clinical frail scale (** ***n*** **)**	
Very fit	0
Well	0
Managing well	2
Vulnerable	4
Mildly frail	2
Moderately frail	3
Severely frail	0
**Cognitive impairment**	
Mini-Cog score < 3	3
Polypharmacy, person (*n*)	9
Falls in last, year (*n*)	5
**Professionals characteristics**	***N*** = **11**
Age, years (mean ± SD)	43 ± 5
**Sex**	
Female (*n*)	9
Male (*n*)	2
**Professions (** ***n*** **)**	
Civic center manager	1
General practitioner	2
Geriatrician	2
Healthcare manager	2
Nurse	1
Physiotherapist	3
Professional experience, years (mean ± SD)	15 ± 4

IQR, interquartile range; SD, standard deviation; SPPB, short physical performance battery. Polypharmacy was defined as the use of more than five drugs.

**Figure 1:**
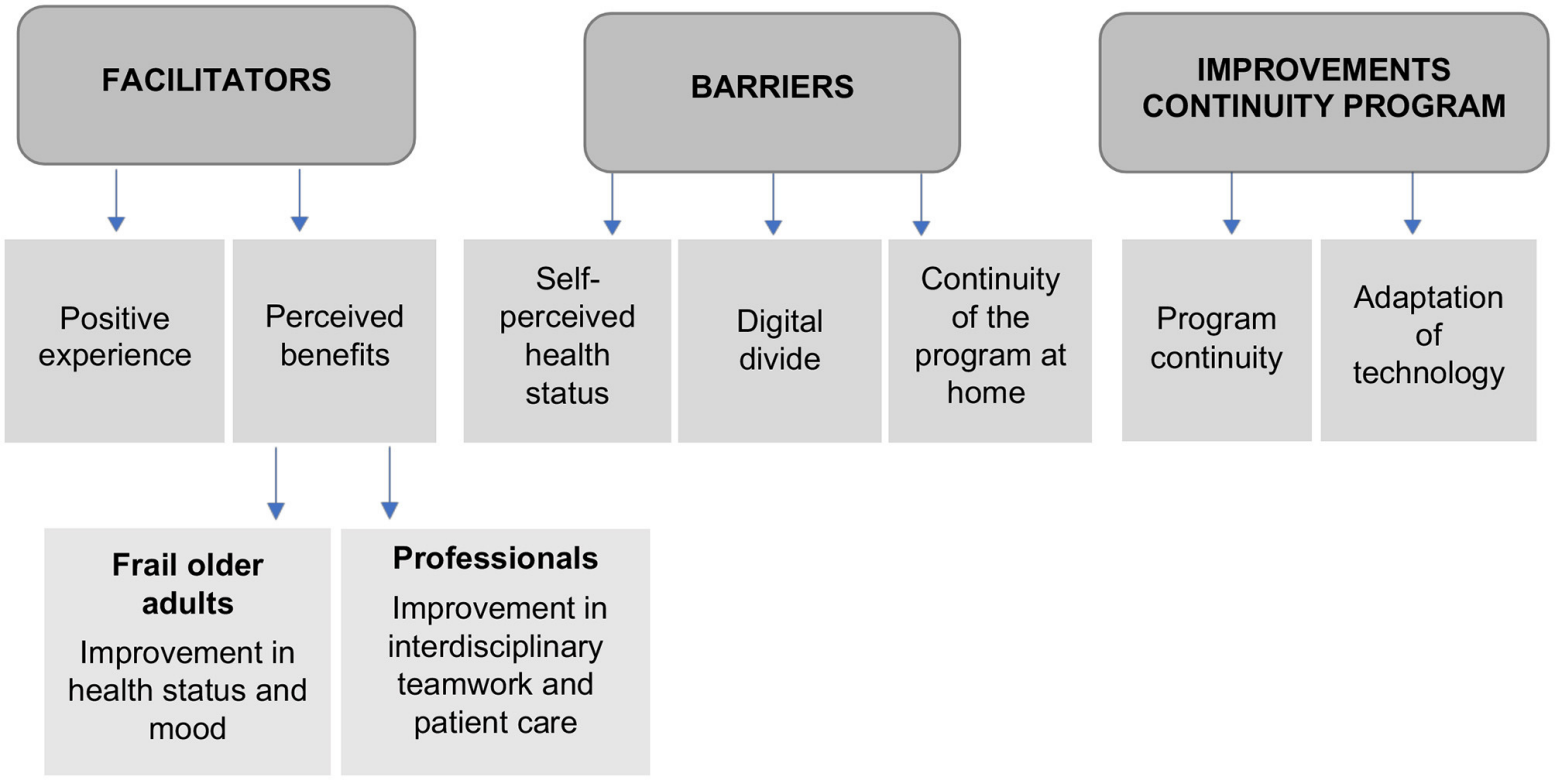
Themes and sub-themes.

The results have been structured into three themes: Facilitators, barriers, and elements of improvement for the continuity of the programme and seven sub-themes shared by users and professionals: positive experience, perceived benefits (at the physical-emotional level and improvement in interdisciplinary work and patient care), self-perceived health status, digital divide, continuity of the programme at home, programme continuity, and adaptation of technology (Figure 1).

### Theme facilitators: Programme experience and benefits obtained by the FOA and professionals

The experience was perceived as very positive for both the FOAs and the professionals. FOAs expressed feeling good throughout their participation in the programme. It was feasible, given that the instructions for performing the exercises were clear, and adapted to each user and the different levels of difficulty or impediment.

“*Next day I feel better than previous. Not only today, no, no, the day after. I” 'm really happy about it. FOA1*.

“*The majority of them, also feel a functional improvement, and they liked it!” PROF4*.

“*…in general it was very nice, very positive* (related to the +AGIL Barcelona programme) *(…) because people feel very happy.” PROF11*.

In addition, the interpersonal relationships created during the programme were valued very positively. It was an incentive for FOAs, a positive obligation, and a motivation to socialize. A close relationship between FOAs and professionals favored this.

“*…socialization, the fact of sitting with more people, the fact of leaving the house, (...) is much higher than being able to do things at home.” PROF3*.

“*Here you participate, you meet one, another one, it is the fact you go out home, to come here and come back, it is very different.” FOA2*.

Thus, both agreed to highlight the perceived benefits on health and quality of life. The FOAs mostly agreed on the perceived benefits of physical exercises, such as improving mood and feeling more agile. They also reported improving their ability to move, balance, and posture, which offered them more security when walking, an essential aspect for those with a history of falls.

“*We previously detected and delayed disability and improved older people's quality of life. Therefore, for me it is the key to this type of implementation.” PROF7*.

“*…it gave me more security for everything, to walk, to move.” FOA1*.

Professionals valued the multidisciplinary approach and teamwork. The programme's success consisted of the team's communication and integration. There was no professional intrusiveness in the teams but they shared criteria from different perspectives, emphasizing the satisfaction of all parties. The comprehensive geriatric assessment was very useful and practical. It provided a comprehensive and holistic vision and allowed the complexity of care to be incorporated into the FOA throughout the intervention, especially in adapting the intervention to each FOA. In general, the professionals perceived that the interconnection between services (primary care and geriatric care) and an expert multidisciplinary health team was essential and beneficial.

“*…here those of us who are here know about exercise, we know about pathology, we know about aging, we connect very well and we create a multidisciplinary team for the whole that we generate together.” PROF9;*

“*…it is very necessary this connection between services, because sometimes we thought “no, that thing is not mine because I'm more in the social part,” at the end we all end up having an impact ways[sic] and therefore it is very necessary to have this connection.” PROF11*.

For the FOAs, the presence of the physiotherapist was one of the most motivating elements. Thus, they appreciated his individual attention, the pleasant atmosphere, the varied exercises, explanations, and patience.

“*I met he* [physiotherapist], *who did not knew[sic] him and I liked him very much, really. I liked him very much and then we started doing what we had to do. And I liked him. I liked the way he talked to us, it seemed like no, that he wasn't looking at you, but he was watching you.” FOA10*.

“*Very nice, treats you well, explains things to you, makes jokes.” FOA7*.

### Theme barriers: Digital divide, the health status of users, and monitoring of the programme at home

The incorporation of digital technology to perform physical exercise online due to COVID-19 was developed with difficulties. FOAs showed low use of online digital technologies, combined with a low predisposition and a strong opposition to the use of technologies due to the lack of skills with devices and apps.

“*It is more pleasant to stay with a group of people than with mobile phones.” FOA5*.

“*I say more than 70% are patients that don't have the technologic skills that we have.” PROF5*.

The closest or most helpful device for them was the mobile phone; some used WhatsApp for video calls, which did have positive aspects as a complement, support, and possible motivation for the programme. However, some FOAs indicated that they would be more positively disposed toward technology if it could be made more accessible to use and if they could have technology support at home to guide them. Finally, FOAs identified their personal devices as very basic, with few applications; consequently, it did not allow them to use a high range of possibilities.

“*…I have a mobile only to call my sons when I needed or when they need me.” FOA2*.

“*Yes, with WhatsApp it was easy for me.” FOA9*.

“*I said no mobiles and tablets, I said no, I couldn't use it and I will get the device and don't use it. However, I learned how to use it step by step.” FOA8*.

The professionals indicated that the FOA presented difficulties in managing the technology. In general, the use is complex for them; they do not know its usefulness, have low learning capacity, are unaware of their digital divide, and prefer face-to-face to socialize, especially after COVID-19.

“*I say more than 70% are patients that don't have the technologic skills we have.” PROF5*.

“*…some of them didn't know or did not answer the call.” PROF10*.

“*Then the majority said yes initially* (at the video call exercise)*; however, when they were in group they said “I prefer to come here, to be in touch, to talk with colleagues, to go to the place we meet,” all this has limited the digitization process.” PROF6*.

The self-perceived health status and expectations were barriers perceived by both professionals and FOAs. In particular, the health status because they recognized their physical limitations or comorbidities. They highlighted aspects such as osteoarticular pain, fatigue, fear of falling, and self-perceived frailty.

“*I come here also for health. During last years I got three, four or five surgeries and I did not have energy.” FOA6*.

“*What I have the most is vertigo, then I have the chair next to me to lean on for a moment.” FOA1*.

In addition, isolation due to COVID-19 has increased the lack of mobility and created more difficulties in moving.

“*…many people benefit from the intervention and also there are people that sometimes surprise us, because they used a trolley walk or they had a very reduced mobility and after, they have improved and of course, these people have lost a lot with the COVID.” PROF4*.

Frail older adults also report problems reconciling personal and family activities with attendance at the programme.

“*…he said “I could not come because my husband or my wife, I have to prepare the meal at that time and in the afternoon I have to accompany him and I won't be able to go, not because I don't want to.” PROF11*.

The professionals identified barriers related to the expectations and lack of awareness of the FOAs, who tend to confuse the programme with physical therapy sessions, or sometimes their initial attitude could be reticent because they had signed up due to the prescription of the primary health professional who referred them or his family.

“*Sometimes when I think there is a lack of communication with the referral team, (…), that patients come a little confused, that they don't know why they come” PROF6*.

Concerning the continuity of the programme at home, carrying out instructions at home was perceived as a barrier by FOAs and professionals due to its low adherence. The FOAs expressed laziness and lack of motivation to exercise alone at home, and once their exercise programme ended, the practice of physical exercise decreased. Therefore, some FOAs tried to maintain the continuity of physical activity in community services.

“*…at home there is no obligation. At home is your house and you do what you want.” FOA1*.

“*…he gave me the exercise shit to do it at home, I do it… sometimes. From time to time, when I'm not lazy. Now, yes here* (in the center)*, yes, I do.” FOA3*.

### Theme improve elements: Continuity of the program and technological adaptation

The continuity of the programme was a prominent topic for both the FOAs and the professionals. Both proposed to increase the number of weeks of the programme or exercise sessions and/or their duration. In addition, they offered long-term follow-up and the option to repeat the programme in future to avoid losing the benefits gained.

“*I thought it is a very good programme, in general. It must be established in every primary care center and more sessions would be better.” PROF10*.

“*Perhaps a good thing should be to have more than ten sessions, (…) and more time.” FOA1*.

The FOAs proposed adding equipment for the exercises and maintaining the social and face-to-face activity. However, the majority stated that the programme was perfect for them. They would not change anything about the activities or exercises, or in their planning, and they valued the figure of the physiotherapist.

“*It was not the type of exercises you say “ups, I can't do it.” No, no, very well.” FOA10*.

“*…the way he explains you understand. And if someone didn't understand, he has a lot of patience to explain to him what he had to do. Because there were quite a few of us and other people… Like me, it's a bit hard for us to talk.” FOA11*.

For the professionals, it was essential to incorporate new community spaces to carry out the sessions and to continue with the personalized and proactive treatment given to the FOAs.

“*But the most important is not only it continuous, is that have to expand and escalate to the rest of the community.” PROF4*.

“*Yes, the visit goes a long way, because really having the time we have to assess a patient as it is done, I think the patient appreciates it a lot….” PROF5*.

It was also proposed to shorten the initial assessment, encourage feedback between professionals and FOAs, and improve coordination between teams by relocating functions.

“*… that improvements have already been implemented with respect to synthesizing the assessment a bit.” PROF2*.

“*In this way, I think that giving regular feedback about the results, as with everything, can help to get confidence in the programme and consolidate it.” PROF10*.

There was a discrepancy between FOA and professionals regarding the importance of resolving the use of technology in the programme. On the one hand, FOAs do not feel this need and dismiss it as a resource, considering that they cannot acquire the necessary technological skills. On the other hand, the professionals valued the usefulness that the use of technologies could represent to give continuity to the programme, proposing a new figure in the team that teaches and is an active help in solving problems with digital applications.

“*But I'm very clumsy with the mobile. I have it for 1 year….” FOA1*.

“*It favors adherence* (use technologies)*, it is clear that sustainability must be sought, and that is why we thought that technology could be one of them. And in this sense (…), learning will be required and, therefore, well, if we were not born technological, someone must teach us.” PROF11*.

“*Many of the people that are on the presential sessions can't do this using a screen.” PROF9*.

## Discussion

The +AGIL Barcelona programme findings were facilitators (positive experience and perceived benefits), barriers (self-perceived health status, digital divide, and continuity of the programme at home has a positive experience), and improvements in the continuity of the programme (programme continuity and adaptation of technology). In addition, all the participants felt satisfied, highlighting aspects such as interpersonal relationships and social contact, face-to-face sessions guided by a physiotherapist, and the functional improvement achieved. Some difficulties were in response to the self-perception of frailty, the need for technological support, and continuing the exercise programme at home.

The +AGIL Barcelona programme was a very positive experience for everyone involved, with different perceptions of the benefits. The participants perceived direct benefits to their health and wellbeing, and the professionals experienced an improvement in the quality of care provided to their users. Both views complement each other and go in the direction of the objectives set by the programme: person-centered design, with a multifactorial strategy, close to the person and built-in integrated care model, involving primary care, geriatrics, and community resources (24).

Person-centered care is an essential aspect of care for older people and is a guiding principle of this programme “Person-centered care” means that individuals' values and preferences are elicited and, once expressed, guide all aspects of their healthcare, supporting their realistic health and life goals (25). Even so, the healthcare reality makes it difficult to implement programmes based on the preferences of the FOAs (25), and, in this sense, it should be noted that +AGIL Barcelona is perceived as a beneficial intervention adapted to the preferences and needs of the FOAs with good healthcare and interprofessional integration. The source of motivation for the participants was the improvement in their health and fitness, changes they had noticed when they performed the exercises. There is evidence to support this result (26). Other aspects that contributed to the positive experience of the programme were as follows: (1) the positive experience that included participation in the face-to-face sessions, (2) sharing the experience with peers, (3) functional and emotional improvement, and (4) social contact. These factors, especially those related to the social aspect of the activity, have been reported previously (27, 28).

The individualized exercise plan was a strong facilitator due to various factors such as attendance, social interaction, individualized prescription adjusted to each participant, and close supervision with a specialized physiotherapist were key points for FOAs to feel capable of performing physical exercise amongst their peers. Previous studies report that the programme's characteristics (individualization, scientific correction, and limited duration) are fundamental to achieving higher levels of adherence to exercise programmes in older adults. These characteristics are similar to the facilitators found in our programme (28, 29).

On the other hand, it has also been reported that reinforcement with peer stimulation, fun during the activity, social aspects driven by performing group activities amongst peers, and the supervision of health professionals are key points to motivate older people to exercise (27, 29, 30). The presence of an expert and multidisciplinary team of geriatricians and physiotherapists integrated into the primary healthcare team is a facilitator of the FOAs care process, resulting in benefits for all those involved. Previous studies highlight the importance of multidisciplinary team intervention to reinforce the FOA's participation and compliance concerning physical activity and healthy habits (29).

In the review by Franco et al. (9), it is stated that the barriers to the participation of older adults in physical activity programmes are as follows: lack of social support, previous sedentary habits, conflicting priorities, accessibility problems, and apathy. Another important barrier is the belief that age-related decline is inevitable and impossible to reverse without the ability to perform the physical activity due to self-perceived frailty. In accordance with this review, in our study, barriers related to self-perception of frailty, comorbidities, pain, conflicting priorities, and fear of falling due to climatic factors (rain) were reported as barriers. However, no lack of social support, apathy, or accessibility problems was reported.

These barriers were addressed (resolved) by developing an individualized programme based on the comprehensive geriatric assessment in an environment close to the FOAs, such as its primary healthcare and community center. The FOAs indicated that their self-perceived health and frailty status were barriers. However, they also indicated that conducting group sessions with people in the same condition, guided by the physiotherapist with exercises adapted to their abilities, was essential for their self-confidence and acceptance that they could perform physical exercise despite their baseline condition.

Finally, an important point to highlight is the use of technology by the participants; they often discard it before trying to use it, or it is challenging for them to use and discard it. This fact has conditioned their assessment of the programme's continuity and technological adaptation. Similar opinions have been reported in other studies (31). There is a recent report on the most common topic barriers to adopting mobile applications for health-related interventions amongst older adults: being unaware of the existence of mobile health applications, lack of technological skills, lack of perceived ability and time, absence of professional involvement, and violation of trust and privacy, many of which were mentioned by the FOAs in this study (32). This topic is a difficult barrier to overcome. There is no single solution path; however, the proposals indicated by FOA and professionals align with what is reported in the literature, e.g., simplifying the technology to the user (33, 34) and receiving education and support for its use (35).

### Strength and limitations

One of the programme's strengths is the high participation of both older adults and professionals. In particular, it highlights the presence of professionals who care for FOA from different areas of the health and social system, thus making their perspective known throughout the care continuum. The main limitation of the current study is that participation in the focus groups was voluntary, which could have resulted in selection bias. This was represented by only one FOA participant agreeing to hold an individual interview, although a broad invitation was made. A possible explanation could be attributed to the difficulty of social interaction generated by the pandemic. On the other hand, this study was carried out in a post-pandemic situation. Therefore, the transferability of the results may be limited.

## Conclusion

The +AGIL Barcelona programme had a positive impact on all the participants. Direct benefits for their health and physical and emotional wellbeing were perceived. The development of interpersonal relationships and social contact managed to develop self-confidence and acceptance to perform physical exercise despite the baseline condition and their self-perception of frailty. There was an improvement in the quality of care due to multidisciplinary teamwork. Some barriers were overcome by a complex person-centered intervention that included a first comprehensive geriatric assessment and guided group sessions of adapted exercises by an expert physiotherapist. The +AGIL Barcelona programme is a complex intervention that requires multiple stages for its implementation and sustainability. This study provides key information to adapt and consolidate community-integrated care programmes.

## Data availability statement

The original contributions presented in the study are included in the article/supplementary material, further inquiries can be directed to the corresponding author.

## Ethics statement

The studies involving human participants were reviewed and approved by Clinical Research Ethics Committee of the Institut Universitari d'Investigació en Atención Primaria, Jordi Gol. The patients/participants provided their written informed consent to participate in this study.

## Author contributions

Conceptualisation: OC-V, MS-R, LMP, and MI. Methodology: OC-V, MS-R, and JR. Validation: OC-V, MS-R, LMP, JR, JV, and MI. Formal analysis: OC-V, LS-N, MS-R, LMP, JR, JV, and MI. Investigation, writing—original draft, and writing—review and editing: OC-V, LS-N, MS-R, LMP, JR, LS-B, RT-C, FD-G, JV, and MI. Supervision and project administration: MS-R, LMP, and MI. Funding acquisition: MS-R, LMP, JV, and MI. All authors contributed to the article and approved the submitted version.
